# Findings in whole body MRI and conventional imaging in patients with fever of unknown origin-a retrospective study

**DOI:** 10.1186/s12880-020-00493-0

**Published:** 2020-08-07

**Authors:** Anoshirwan Andrej Tavakoli, Miriam Reichert, Tanja Blank, Dietmar Dinter, Sabine Weckbach, Dieter Buchheidt, Stefan Oswald Schoenberg, Ulrike Attenberger

**Affiliations:** 1 Department of Clinical Radiology and Nuclear Medicine, University Medical Center Mannheim, Mannheim, Germany; 2 Department of Radiology, German Cancer Research Center (Dkfz), Heidelberg, Germany; 3 Radiologie Schwetzingen, Schwetzingen, Germany; 4 Department of Radiology, University Hospital Heidelberg, Heidelberg, Germany; 5 Department of Hematology and Oncology, University Medical Center Mannheim, Mannheim, Germany; 6 Department of Radiology, University Hospital Bonn, Bonn, Germany

**Keywords:** Fever of unknown origin, FUO, Whole-body MRI, Inflammatory focus, Focus detection

## Abstract

**Background:**

To analyse the influence of whole body (wb)-MRI on patient management compared to routine diagnostic tests in patients with fever of unknown origin (FUO).

**Methods:**

Twenty-four patients with FUO, defined as illness of more than three weeks with fever greater than 38.3 °C, underwent wb-MRI at a 1.5 T MR-system. The MR-protocol consisted of the following sequences: axial T1 VIBE, coronal T2-TIRM and a coronal echoplanar diffusion weighted sequence (overall acquisition time 29:39 min:s). Furthermore, laboratory findings, chest-x-ray, abdominal ultrasound, CT-scans and/or PET-CT scans were evaluated and compared to the wb-MRI findings in regard to treatment changes.

**Results:**

Wb-MRI yielded a correct diagnosis in 70% of the patients. In 46% the inflammatory focus was exclusively detected by wb-MRI. Focus detection by wb-MRI led to a subsequent change of the clinical management in 92% of the patients. In 6 patients both a wb-MRI and a PET-CT were performed yielding the correct diagnosis in the same 4 of 6 patients for both imaging modalities.

**Conclusions:**

Wb-MRI appears to be of value in the evaluation of FUO patients, allowing for optimized treatment by increasing diagnostic certainty. Due to its lack of nephrotoxicity and ionizing radiation it may be preferred over standard imaging techniques and PET-CT in the future. However, given the low number of patients in our trial, further prospective studies have to be performed to confirm our results.

## Background

Fever of unknown origin, defined as body temperature of more than 38.3 °C for more than three weeks without an infectious focus after three outpatient visits or three days of inpatient clinical investigations, still imposes a time and resource-consuming task for clinicians [1–3]. Even though a timely diagnosis is essential for optimal patient treatment, conventional diagnostic tests often fail to quickly and reliably identify the origin of the fever. Frequently the focus is found late or not at all, thus prolonging hospitalization, increasing the risk of nosocomial infections and raising the overall costs of care [4–6]. With the emergence of PET-CT scans, a powerful tool with high detection rate is available in the FUO diagnostic work-up [7–9]. However due to radiation exposition and generally low availability, the feasibility of deploying PET-CTs in routine assessment of patients with FUO remains restricted.

In the past decade advances in MRI-technology have made the wb-MRI a widely used diagnostic test [10–12]. A whole-body examination is performed without radiation exposition and with low incidence of allergic side effects in just one sitting, thus qualifying as a widely deployable examination, suitable even for children [13, 14]. Wb-MRI already represents an established alternative to existing algorithms in diagnosing cardiovascular diseases and it has been shown to have prognostic value in patients with diabetes mellitus and rheumatic disease [15–17]. The diagnostic value of wb-MRI in finding the cause of a FUO, however, has to-date not been described in a clinical study.

Here, we hypothesize that wb-MRI is more feasible than conventional examinations in the diagnosis of FUO. To address this question, we conducted a retrospective clinical study in 24 patients with FUO who had received a wb-MRI and compared the subsequent changes of clinical management to those following conventional diagnostic tests and/or PET-CT scans.

## Methods

The study was designed as a retrospective evaluation patient data with FUO who received a prospectively plannend wb-MRI between October 2009 and September 2011. The wb-MRI was performed to potentially reach a diagnostic accuracy of wb-MRI to conventional test. FUO was defined as an illness of more than three weeks with body temperature greater than 38.3 °C and no diagnosis after either three outpatient visits or inpatient status for more than three days. All patients fulfilling these criteria were schedulded to receive a wb-MRI at our institution by the attending physician in the stated period of time. Wb-MRI data sets were included only if a defined set of MRI sequences (see below) was present. The study was approved by the institutional review board and all patient data were anonymized before usage. Written consent was obtained from each patient. There are no known conflicts of interest associated with this publication and there has been no significant financial support for this work that could have influenced its outcome.

### Patient data

The selected group of 24 patients consisted of 16 men and 8 women (Table 1). The wb-MRI was conducted after a mean hospitalization of 10 days. Clinical data regarding the age, gender, biochemical and hematological diagnostic tests, hospitalization and clinical evaluation were asserted. Further, all clinical diagnostic tests 21 days prior to and 21 days posterior to the wb-MRI including chest-x-ray, ultrasound, CT and/or PET-CT were evaluated for either providing the cause or an indication of the FUO. All images were prospectively evaluated by the radiologists of the clinical routine, which consisted of a resident and the attending radiologist. Subsequently, the changes in clinical management after wb-MRI were analysed and compared to treatment changes after conventional tests. This change of clinical management was defined as any new therapy, an alteration of an existing therapy or any new targeted diagnostic procedure and was carefully reviewed from patient data to be directly related to the imaging reports. All patient data are available from the authors upon reasonable request.

**Table 1:** Patient demographics

age [years]	mean ± SD	54 ± 21
	median	58 (7–81)
gender	male	16
	female	8
Neutropenia	Yes	1
	No	23
CRP (mg/l)	mean ± SD	102 ± 89
focus category*	hepatobiliary	3
	musculoskeletal	2
	respiratory	6
	cardial	5
	enteropathic	2
	other	4
	none	4
Underlying disease	Yes	14
	No	10

*Note: Two patients had two infectious foci

Neutropenia was defined as < 1000/μL leucocytes

*CRP* C-reactive protein

*SD* Standard deviation

### Imaging

Whole body MRI (Magnetom Avanto 1.5 Tesla, Siemens healthineers, Erlangen, Germany) was conducted with a standardized FUO-protocol consisting of a pre-contrast axial T1 VIBE of the head, whole body STIR and echoplanar diffusion weighted (EPI-DWI) sequences in coronal orientation from head to feet, an axial EPI-DWI sequence of the cranium, an axial T1 VIBE post-contrast from the skull base to feet, and a separate T1 VIBE post-contrast of the head (Table 2). After conducting the STIR and EPI-DWI sequences and the pre-contrast T1 VIBE of the head, 0.5 M Gadoterate meglumine (Dotarem, Guerbet, Sulzbach, Germany) and 40 ml isotonic NaCl were administered at an injection rate of 1.5 ml/s. The total duration of the examination was 29:39 min:s.

**Table 2:** Scan parameters

Sequence parameters	STIR	EPI-DWI head	EPI-DWI	T1 VIBE CE	T1 VIBE head CE
Echo Time [ms]	105	76	76	2.38	10
Repetition time [ms]	5220	3900	5300	5.46	450**
FoV read [mm]	500	379	459	500	230
FoV phase	100%	100%	100%	68.75%	87.50%
Matrix	512 × 512	192 × 192	192 × 192	320 × 320	256 × 202
In plane resolution [mm^2^]	0.98 × 0.98	1.97 × 1.97	2.39 × 2.39	1.56 × 1.07	0.90 × 1.0
Slice thickness [mm]	6	4	5	3	5
Orientation	coronal	axial	coronal	axial	axial
Merged stacks	4	1	4	8*	1
Range	head to tow	head	head to tow	skull base to tow	head
Bandwidth [Hz/px]	181	1628	1628	260	90
b-values [s/mm^2^]	–	50/400/800	50/400/800	–	–
Averages	2	2	3	1	1

*CE* contrast enhanced

*FoV* field of view

*STIR* short tau inversion recovery

*VIBE* volumetric interpolated breath-hold examination

*EPI* echoplanar imaging

*DWI* diffusion weighted imaging

* Dependent on patient height

** T1 VIBE of the head was performed pre- and post-contrast. The pre-contrast repetition time was 490 ms

### Data analysis

Wb-MRI data analysis was conducted by two experienced radiologists with special attention for infectious foci. Both readers were informed about the existing fever and all available wb-MRI sequences were evaluated to reach a diagnosis. Readers were not independent and there was inter-reader consensus. Next, the relevance of the radiological report for final clinical diagnosis and for the change of clinical management was evaluated.

## Results

**Fig. 1:**
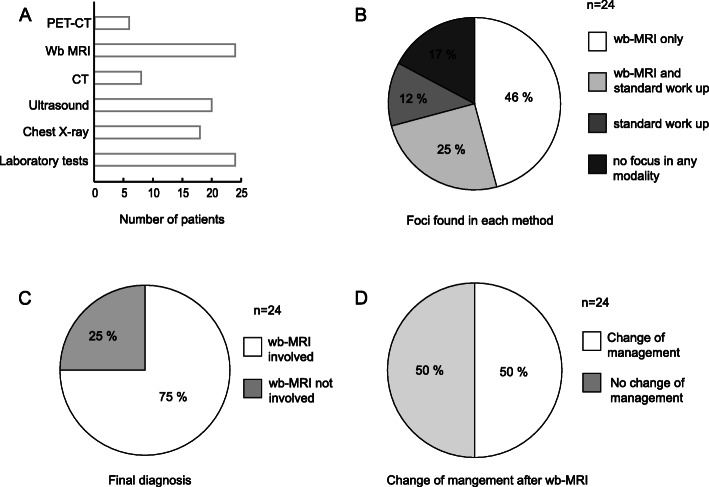
Summary of wb-MRI performance versus standard clinical work-up and PET-CT in diagnosing infectious foci in patients with FUO. A bar graph of diagnostic tests **a** shows the number of patients receiving each respective clinical test. **b** Altogether 46% of infectious foci were found by wb-MRI alone compared to standard clinical tests. PET-CT found the same 4/6 infectious foci as wb-MRI in patients on whom both examinations were performed (not shown in the graph). Note that the infectious foci diagnosed by standard diagnostics alone (12%) were all cases of endocarditis revealed by cardiac ultrasound. In 50% of patients a change of clinical management immediately followed the wb-MRI **c** and in all cases in which a focus was found in any imaging modality (75%) the wb-MRI was directly involved in the final diagnosis **d**

Twenty-four patients (mean age 54 ± 21 years, range 7–81 years) with fever of unknown origin completed the requirements of the study (Table 1). All clinical standard examinations that were performed within 21 days prior and 21 days posterior to performance of the wb-MRI were considered for analysis. The mean hospitalization duration of the patients was 23.2 ± 16.5 days. All patients had complete hemograms with more than half of the cohort displaying leucocytes different from the norm, with one patient in neutropenia (leucocyte level of < 1000/μL). Almost all patients had elevated levels of C-reactive protein. The bar graph in Fig.1a provides a summary of the total number of diagnostic tests performed on the cohort of the 24 patients.

### Wb-MRI detects inflammatory foci more reliably than standard clinical work-up

The origin of the FUO of 83% patients could be detected by all diagnostic tests combined while 17% patients remained without a defined inflammatory focus as the source of the fever. Wb-MRI was performed in each patient after a mean of 10 days after initial hospitalization. In 79.2% of patients a pathology was detected by wb-MRI and the detection rate for inflammatory foci as a cause of the FUO was 71%. For almost half of the patients solely the wb-MRI provided a focus (Fig. 1b), when compared to conventional diagnostic tests. In three quarters of the cohort wb-MRI had a direct influence on the final clinical diagnosis (Fig. 1c). In half of the patient cohort the report of the wb-MRI resulted in an immediate change of clinical management with a subsequent change of therapy in almost all of these patients, which would not have been conducted without the wb-MRI (Fig. 1d).

### Comparison of conventional diagnostics to wb-MRI

**Fig. 2:**
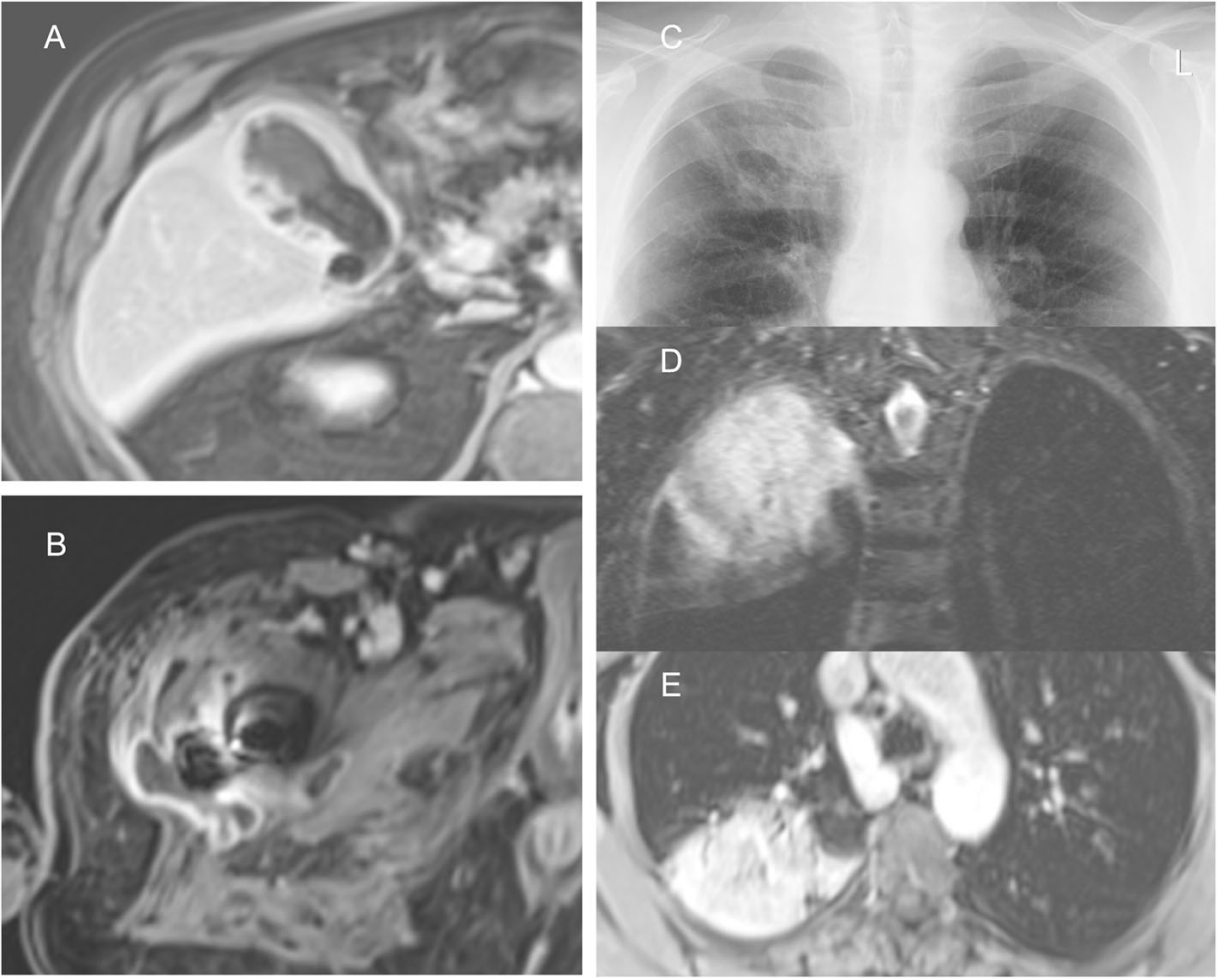
Infectious foci found by wb-MRI in patients with FUO. Axial contrast enhanced T1-VIBE image of a 81-year old-patient **a** shows gallbladder wall thickening, contrast enhancement and lithiasis as signs of subacute cholecystitis which had eluded diagnosis by abdominal ultrasound. In another patient, an 88-year-old female, **b** the contrast enhanced T1-VIBE displays a perifemoral intramuscular abscess formation as infectious focus which remained occult after standard clinical work-up. Conventional chest radiography **c**, coronal T2-STIR sequence **d** and axial contrast enhanced T1-VIBE **e** of a 67-year-old patient with right upper-lobe pneumonia. The radiography shows discrete reticular opacities in the right upper zone suggesting pulmonary infiltration while T2-STIR and contrast enhanced T1-VIBE prove right upper lobe pneumonia as infectious focus. The radiography was taken 1 day prior to the wb-MRI

The diagnostic value of the wb-MRI was not only superior to all standard examinations but also to all standard diagnostic tests combined. Eighteen patients received chest X-rays. In 1/18 patients a pneumonic infiltration was found as the infectious focus by both X-ray and wb-MRI (Fig. 2c-e). In the remaining 17/18 X-ray examinations the infectious focus was not found, whereas the wb-MRI found the cause of the FUO in three additional cases within the thorax and five additional cases outside the thorax.

Targeted CT on organ regions where a focus was clinically suspected was performed on 8 patients without previous CT scans. 2/8 of the CT examinations identified the underlying cause of the FUO, while 1/8 CT provided an indication for later diagnosis. In comparison, the wb-MRI found the inflammatory focus in the same 2/8 patients, could establish a diagnosis for the focus in the 1/8 the CT report had suggested and additionally found the focus in 3/8 more patients, in which the CT failed to establish any diagnosis. In 2/8 cases neither the targeted CT nor the wb-MRI found the focus.

A total number of 29 sonographical examinations were performed in 20 patients, 12 cardiac ultrasounds, 15 abdominal ultrasounds and 2 vascular ultrasounds. 5/12 cardiac ultrasounds either found the focus or gave an indication to the focus. 0/15 abdominal ultrasounds found the infectious focus, but 2/15 gave an indication for later diagnosis. 1/2 vascular ultrasounds established an infectious focus. Altogether 6/29 examinations diagnosed a focus with 2/29 examinations providing indications for the later diagnosis. 21/29 examinations had no result regarding the FUO. Notably, 5/8 (40%) examinations that either found the focus or gave an indication were echocardiographic ultrasounds. Of these 5 cases detected by cardiac ultrasound, only 2/5 were detected by wb-MRI, whereas it was negative in 3/5 cases. These were the only cases, in which the standard work up found a focus not detected by wb-MRI, likely because of artifacts caused by cardiac movements.

**Fig. 3:**
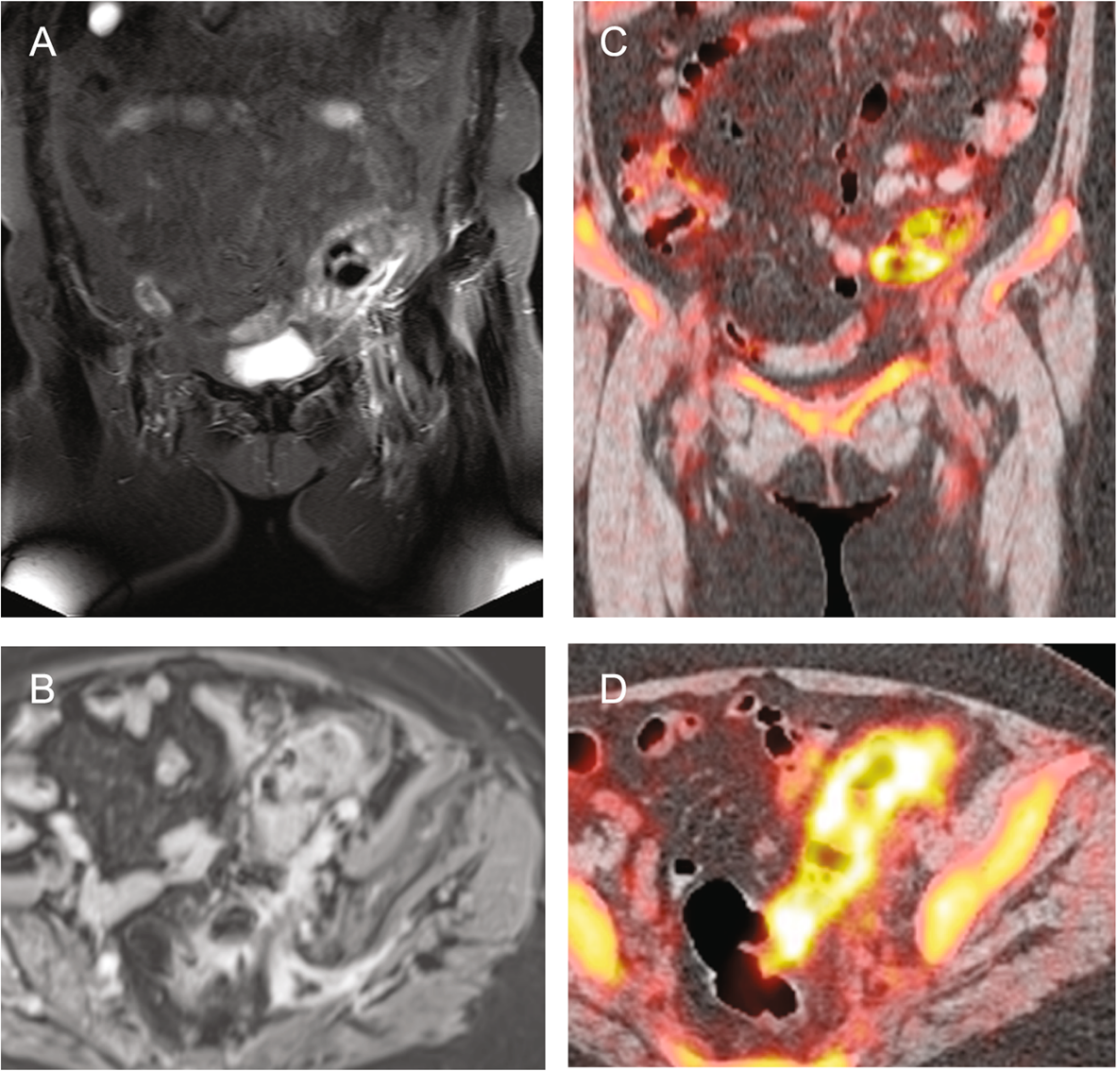
76-year-old patient with FUO suffering from occult sigmoid diverticulitis who underwent both wb-MRI and PET-CT. Coronal T2-STIR **a** and axial contrast enhanced T1-VIBE **b** images reveal sigmoid wall edema and perifocal fat-stranding in accordance with sigmoid diverticulitis. Coronal and axial FDG-PET-CT **c**, **d** display elevated tracer uptake of the sigmoid as sign of acute inflammation

**Fig. 4:**
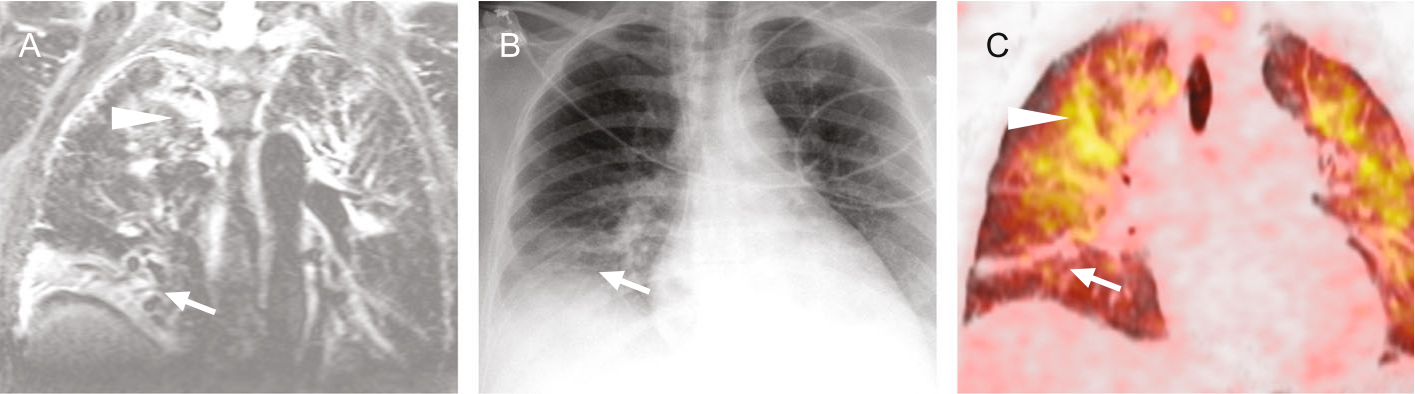
56-year-old patient with pulmonary infiltrations as infectious focus. Juxtaposition of coronal T2-STIR **a**, conventional radiography of the chest **b** and coronal PET-CT **c** in chronological order. One day elapsed between each image. Note that the middle lobe atelectasis (arrow) resolves over time, while the paramediastinal infiltrations (arrow head) increase. The conventional radiography **c** shows signs of the atelectasis in the right lower zone but no clear sign of the paramediastinal pulmonary infiltration

Altogether 6 of the 24 patients received both a PET-CT and a wb-MRI, with both methods finding the same inflammatory foci in the same 4 patients, while the other two patients remained without a macroscopic focus. The resulting detection rate for both methods for these 4 cases was thus 66.7%. Figure 3 shows a case of a 76-year-old patient who received both modalities which found an occult sigmoid diverticulitis to be the cause of the fever. In a patient with lung infiltration (Fig. 4), wb-MRI and PET-CT reliably detected the same infectious focus, while the chest radiography failed to show a clear sign of the infiltration in the right apex.

## Discussion

Up to date only few studies evaluating the clinical value of wb-MRI in the diagnosis of fever of unknown origin exist [18, 19], leaving the relevance for its diagnostic work up undefined. Our retrospective study of 24 patients portrays findings regarding the diagnostic value of wb-MRI in patients with fever of unknown origin and compares the diagnostic success after whole body MR scans to standard diagnostic methods and/or PET-CT.

Although wb-MRI has become a widely accepted method in whole body imaging within the last decade [20], it has not yet been implemented in the diagnostic routine of FUO. Currently the role of MRI in FUO diagnostic is usually limited to answering organ-specific questions [21]. With wb-MRI there is a non-invasive, high-resolution method at hand, allowing a comprehensive assessment of the whole body in only one examination in the reasonable duration of 29:39 min:s. Notably, in our study the wb-MRI report was directly involved in finding the final clinical diagnosis in 75% of the cases while 17% of the fever origin remained unknown entirely. Wb-MRI further stated the exact location of the inflammatory focus in 70.8%, while the conventional combined tests only yielded a sensitivity of 25%. Wb-MRI was directly responsible for an immediate change of clinical management in 50% of the cases. It must be emphasized that as many as 50% of the patients would have remained undiagnosed and would not have received the appropriate change of clinical management, if the wb-MRI had not been performed.

The most important limitation of the wb-MRI as a method of FUO detection was the low rate of endocarditis detection. In our study this was reflected by the three patients, whose endocarditis as infectious focus could only be detected by cardiac echography, but not by wb-MRI, likely due to artifacts caused by cardiac motion, thus limiting the diagnostic value. These results suggest that in case of a suspected endocarditis or negative results in the wb-MRI an additional cardiac echography should be conducted.

Wb-MRI appears to even be comparable to PET-CT in establishing a focus: In the most recent meta-analysis examining the value of PET and PET-CT in FUO detection, the mean rate of examinations contributing to the final diagnosis was 48% (range between studies 11–69%) [7], while wb-MRI stated the exact location of the focus in 66.7% in our study, which is on the upper end of the range of the diagnostic accuracy of PET and PET-CT. Further support of the hypothesis that both methods may be comparable is shown by the six cases in our study where both wb-MRI and PET-CT were performed. Both methods found the same inflammatory focus in the same 4 of 6 patients, while two patients remained without macroscopic cause of the fever.

There are limitations to this study. First, there was a low total number of patients included in this study because of the relatively rare occasion of FUO due to the defining criteria. The low number makes complete statistic tests of the data infeasible, and thus no statistically valid conclusions can be drawn from the study. A second limitation to the study is the fact that only a small number of patients concurrently received a PET-CT as a gold standard to exclude or confirm a focus, which however would be necessary to quantify the performance for wb-MRI and calculate diagnostic test parameters. Once the wb-MRI already had established an inflammatory focus, usually treatment was started immediately rendering a further PET-CT unnecessary and a further delay unethical. However, it has to be noted that the patients that remained without clear focus could be discharged from the hospital with spontaneous recovery from FUO without further treatment or complications. Third, due to the restrospective nature of the study we cannot fully exclude biases with regard to the retrospective evaluation of the change in clinical management after the examinations had taken place. Further studies would require a larger prospective cohort, including a PET-CT for each patient as ground truth to directly compare the value of wb-MRI to PET-CT.

## Conclusion

Our results indicate that wb-MRI may be a feasible investigation in FUO diagnostic in patients with negative radiograph and ultrasound instead of focused CT or PET-CT. To confirm our results, a prospective study with a larger cohort is required, optimally performing additional PET-CTs for each patient to directly compare the value of wb-MRI to PET-CT.

## Data Availability

The datasets generated and analysed during the study are not publicly available due to patient privacy, but are available from the corresponding author upon reasonable request.

## Abbreviations

Wb: Whole body
FUO: Fever of unknown origin
EPI-DWI: Echoplanar diffusion weighted imaging
MRI: Magnetic resonance imaging
PET: Positron emission tomography
CT: Computed tomography
VIBE: Volumetric interpolated breath-hold examination
STIR: Short tau inversion recovery
